# Casein Micelles as Nanocarriers for Benzydamine Delivery

**DOI:** 10.3390/polym13244357

**Published:** 2021-12-13

**Authors:** Nikolay Zahariev, Maria Marudova, Sophia Milenkova, Yordanka Uzunova, Bissera Pilicheva

**Affiliations:** 1 Department of Pharmaceutical Sciences, Faculty of Pharmacy, Medical University of Plovdiv, 15A Vassil Aprilov Blvd, 4002 Plovdiv, Bulgaria; nikolay.zahariev@mu-plovdiv.bg; 2 Research Institute, Medical University of Plovdiv, 15A Vassil Aprilov Blvd, 4002 Plovdiv, Bulgaria; yordanka.uzunova@mu-plovdiv.bg; 3 Faculty of Physics and Technology, University of Plovdiv “Paisii Hilendarski”, 24 Tsar Asen Str., 4000 Plovdiv, Bulgaria; marudova@uni-plovdiv.net (M.M.); sophiamilenkowa@gmail.com (S.M.); 4 Department of Bioorganic Chemistry, Faculty of Pharmacy, Medical University of Plovdiv, 15A Vassil Aprilov Blvd, 4002 Plovdiv, Bulgaria

**Keywords:** benzydamine, casein, biopolymers, nanoparticles, nano spray drying, nano micelles, drug delivery

## Abstract

The aim of the present work was to optimize the process parameters of the nano spray drying technique for the formulation of benzydamine-loaded casein nanoparticles and to investigate the effect of some process variables on the structural and morphological characteristics and release behavior. The obtained particles were characterized in terms of particle size and size distribution, surface morphology, production yield and encapsulation efficiency, drug-polymer compatibility, etc., using dynamic light scattering, scanning electron microscopy, differential scanning calorimetry, and Fourier transformed infrared spectroscopy. Production yields of the blank nanoparticles were significantly influenced by the concentration of both casein and the crosslinking agent. The formulated drug-loaded nanoparticles had an average particle size of 135.9 nm to 994.2 nm. Drug loading varied from 16.02% to 57.41% and the encapsulation efficiency was in the range 34.61% to 78.82%. Our study has demonstrated that all the investigated parameters depended greatly on the polymer/drug ratio and the drug release study confirmed the feasibility of the developed nanocarriers for prolonged delivery of benzydamine.

## 1. Introduction

Over recent decades there has been a growing scientific interest towards the use of naturally occurring materials for drug delivery purposes. This is mainly due to their numerous advantages over synthetic materials, namely biocompatibility, biodegradability, and low immunogenicity [1]. Moreover, natural materials produce non-toxic metabolites, unlike synthetic polymers which can be contaminated with unreacted toxic monomers and crosslinkers [2]. Due to their specific structure and corresponding features, naturally occurring materials such as polysaccharides and peptides are widely used for the formulation of micro- and nanoparticulate drug delivery systems. These carriers can provide controlled and targeted release, thus improving the therapeutic performance of the encapsulated drug and minimizing the risk of side effects [3]. Amongst the potential biopolymers, proteins are preferred as natural drug delivery systems due to the relatively easy preparation processes and production of well-defined structures, which enables surface modification and may provide modified and targeted release [1]. Among proteins, casein (CAS) is considered a suitable biopolymer for the preparation of nanoparticulate drug delivery systems due to its structural and physicochemical characteristics [2].

Casein is a collective term used to define a family of calcium (phosphate)-binding phosphoproteins commonly found in mammalian milk [4]. Casein from bovine milk is composed of four peptides, namely α_s1_, α_s2_, β, and k, which differ in the content of amino acids, phosphorus, and carbohydrates, but they are all amphiphilic in nature [5]. Cysteine amino acid residues that allow the formation of disulfide bonds are found only in the polypeptide chains of k-casein. In general, the peptide surface is negatively charged due to phosphorylation [1]. The lack of secondary structures because of the proline-rich amino acid sequence [6] and the tendency for binding amorphous calcium phosphate cause electrostatic, hydrogen, and hydrophobic interactions, leading to self-assembly of the casein peptides into stable agglomerates known as casein micelles [7]. The inner part of the micelle is composed of α_s1_, α_s2_, and β caseins, whereas the outer layer that stabilizes the micelle contains glycosylated k-casein [8]. Casein micelles exhibit pH-dependent behavior. Their structure tightens when the negative surface charge of casein molecules decreases, and expands with increasing surface charge, which leads to electrostatic repulsion between the molecules [9,10,11]. Given the amphiphilic properties and pH-dependent behavior of casein, and its ability to participate in hydrophobic and hydrophilic interactions, it is clear why this biopolymer has found a place in scientific research as a potential nanoparticle drug delivery carrier.

Various methods have been reported for the preparation of casein nanoparticles for drug delivery, including pH-shifting [12], high pressure homogenization [13,14,15,16,17,18], electrostatic complexation [19], solvent displacement [20], emulsification solvent evaporation [21], and spray drying [22,23,24]. Nano spray drying, a variation of the established spray drying technology used to convert liquids into solid powders, is a relatively new technique adopted for the preparation of nanosized drug delivery systems. The method is based on the use of a revolutionary sprayer developed by the Swiss Büchi Labortechnik AG, which is equipped with a piezoelectric vibrating spray mesh head, allowing the formation of fine droplets, which are dried and electrostatically collected [25]. As a result, spherical submicron structures of particle size below 1000 nm with improved biopharmaceutical behavior are obtained [26,27,28,29,30,31,32,33]. Although spray drying of proteins has been reported in numerous scientific papers [34,35,36,37], no data on nano spray drying of casein have been found in the literature. The technology was therefore a research challenge. For the present study, benzydamine hydrochloride (BZ) was used as a model drug.

Benzydamine hydrochloride is a nonsteroidal anti-inflammatory drug with local anesthetic and analgesic properties for pain relief and treatment of inflammatory conditions of the mouth and throat such as oral mucositis, postoperative sore throat and mucosal ulcers. The mechanism of the anti-inflammatory effect of benzydamine has not yet been fully understood. According to Quane et al. [38], the anti-inflammatory activity of benzydamine may be due to its membrane-stabilizing or inhibitory effect of the synthesis of TNF-α. Unlike NSAIDs, which have acidic properties, benzydamine is a weak base, highly lipid-soluble in its unionized form [39].

According to Beckett et al. [40] and Bickel et al. [41], only a limited amount of weak, basic, lipid-soluble drugs is absorbed into buccal tissue via mouthwash application. The small degree of absorption into buccal tissue is confirmed by the poor systemic availability (5%) [42]. To enhance absorption and thus bioavailability, benzydamine hydrochloride was incorporated into nanoparticles. Due to the specific binding properties and pH-dependent drug release, casein is considered a promising biopolymer for the preparation of benzydamine loaded casein nanoparticles.

The aim of the present work was to optimize the process parameters of the nano spray drying technique for the formulation of BZ-loaded casein nanoparticles. Furthermore, an investigation of the effect of process variables on structural and morphological characteristics and release behavior was conducted.

## 2. Materials and Methods

Benzydamine hydrochloride (Mw 345.87 g/mol), sodium caseinate (from bovine milk) and CaCl_2_·2H_2_O (Mw 147.01 g/mol) were purchased from Sigma-Aldrich (St. Louis, MO, USA). All other reagents were of analytical grade.

### 2.1. Preparation of Blank Casein Nanoparticles and BZ-Loaded Casein Nanoparticles

The blank casein nanoparticles where prepared via coacervation followed by spray drying using nano spray dryer Büchi B-90 (Büchi Labortechnik AG, Flawil, Switzerland), as previously reported by Gandhi et al. [2]. A certain amount of sodium caseinate was dissolved in 100 mL deionized water, previously adjusted to pH 2 with 1M hydrochloric acid. Then, the crosslinking agent CaCl_2_·2H_2_O (2 µL/mL) was added dropwise to the casein solution under high-speed homogenization at 25,000 rpm (Miccra MiniBatch D-9, MICCRA GmbH, Heitersheim, Germany) for 15 min and casein micelles were produced. The obtained nanosuspension was then stirred on a magnetic stirrer at 500 rpm for 30 min) to allow effective crosslinking of casein molecules. Finally, the suspension was spray dried using nano spray dryer Büchi B-90 under the following predetermined conditions: mesh size of 4.0 μm, inlet temperature 40 °C, solution feed rate 50%, spray intensity 70%, drying gas speed 120 L/min, pressure 30 nbar. To study the effect of different formulation variables on the produced particles, 3^2^ full factorial design was applied. Nine batches of formulations were prepared at varied protein and crosslinker concentrations (Table 1).

BZ-loaded casein nanoparticles were prepared following the methodology described in the previous paragraph. Briefly, protein aqueous solution (1.5% *w*/*v*) was prepared by dissolving a certain amount of sodium caseinate in deionized water, previously acidified to pH 2 using 1N hydrochloric acid. Then, BZ was added to the solution, followed by protein crosslinking with CaCl_2_·2H_2_O (2 µL/mL) at a stirring rate of 25,000 rpm. The procedure continued as previously described. Four batches of drug-leaded formulations were developed at varied drug–polymer ratios. The composition of the batches is presented in Table 2.

### 2.2. Characterization

#### 2.2.1. Production Yield, Drug Loading and Entrapment Efficiency

The production yields of the nanoparticles from different batches were calculated using the weight of the spray dried nanoparticles with respect to the initial quantity of the drug and polymer, according to the following equation:

(1) $$\mathrm{Production}\;\mathrm{yield}\;\left(\%\right)=\frac{\mathrm{Spray}\;\mathrm{dried}\;\mathrm{nanoparticles}\;\left(\mathrm{mg}\right)}{\mathrm{Drug}\;\left(\mathrm{mg}\right)+\mathrm{Polymer}\;\left(\mathrm{mg}\right)}$$

Drug loading and drug entrapment efficiency of BZ-loaded CAS nanoparticles were determined spectrophotometrically. BZ-loaded nanoparticles were dispersed into 10 mL previously acidified at pH 2 deionized water and were stirred for 60 min until complete swelling of casein micelles occurred, allowing BZ extraction in the aqueous medium. Then, the blend was centrifuged at 5000 rpm and filtered (0.22 µm, Chromafil^®^, Macherey-Nagel, Düren, Germany). Drug concentration was determined after proper dilution using an Evolution 3000 Pro UV/Visible spectrophotometer (Thermo Scientific, Waltham, MA, USA) at a wavelength of 306 nm. The drug loading (DL) was calculated according to Equation (2), and entrapment efficiency (EE) was calculated according to Equation (3):

(2) $$\mathrm{DL}\;\left(\%\right)=\frac{\mathrm{Amount}\;\mathrm{of}\;\mathrm{drug}\;\mathrm{in}\;\mathrm{the}\;\mathrm{formulation}}{\mathrm{Total}\;\mathrm{amount}\;\mathrm{of}\;\mathrm{nanoparticles}}\times 100$$

(3) $$\mathrm{EE}\;\left(\%\right)=\frac{\mathrm{Actual}\;\mathrm{drug}\;\mathrm{content}}{\mathrm{Theoretical}\;\mathrm{drug}\;\mathrm{content}}\times 100$$

#### 2.2.2. Particle Size Analysis, Size Distribution and Zeta Potential

Particle size of the obtained CAS nanoparticles was determined by dynamic light scattering method using Nanotrac particle size analyzer (Microtrac, York, PA, USA). The system is equipped with 3 mW helium/neon laser at 780 nm wavelength and measures the particle size with noninvasive backscattering technology, performing particle size analysis in the range of 0.8 nm to 6.5 µm. The equipment allows determination of ζ-potential in the range from −200 mv to +200 mV. The samples were prepared by dispersing a small amount of dry nanoparticles in purified water, and the dispersions (refractive index 1.33, average viscosity 0.87 ± 0.05 cP) were stirred on a magnetic stirrer and then analysed for particle size and zeta potential. All the measurements were performed at 25.0 °C at 20-s intervals and were repeated three times.

#### 2.2.3. SEM Analysis

Imaging of the obtained CAS nanoparticles was performed using scanning electron microscopy (Prisma E SEM, Thermo Scientific, Waltham, MA, USA). The samples were loaded on a copper sample holder and sputter coated with carbon followed by gold using vacuum evaporator (BH30). The images were recorded at 15 kV acceleration voltage at various magnifications using DBS (back-scattered electrons) detector.

#### 2.2.4. FTIR Spectroscopy

The samples were evaluated for drug/polymer interactions by Fourier transformed infrared spectroscopy (FTIR). The spectra were collected using a Nicolet iS 10 FTIR spectrometer (Thermo Fisher Scientific, Pittsburgh, PA, USA), equipped with a diamond attenuated total reflection (ATR) accessory, operating in the range from 600 cm^−1^ to 4000 cm^−1^ with a resolution 4 nm and 16 scans. The obtained spectra were analysed with OMNIC^®^ software package (Version 7.3, Thermo Electron Corporation, Madison, WI, USA).

#### 2.2.5. Differential Scanning Calorimetry (DSC)

Thermal analysis of the CAS nanoparticles was performed using DSC 204F1 Phoenix (Netzsch Gerätebau GmbH, Selb, Germany) based on the heat flux principle and cooled with a an intracooler. An indium standard (T_m_ = 156.6 °C, ΔH_m_ = 28.5 J/g) was used for the temperature and heat flow calibration. The samples were hermetically sealed in aluminum sample pans. An empty pan, identical to the sample pan, was used as reference. The measurements were performed under argon atmosphere at a heating rate of 10 °C/min.

#### 2.2.6. In Vitro Drug Release

In vitro release study was carried out by diffusion using dialysis bag. The dialysis membrane (Sigma, MWCO 12,000 Da) was cut into equal pieces (6 × 2.5 cm^2^) and soaked in distilled water for 24 h before use. An accurately weighed amount of nanoparticles (equivalent to 10 mg BZ) was dispersed in 2 mL of PBS buffer (pH = 7.4) and transferred into the dialysis bag. Each bag was placed into a beaker containing 20 mL dissolution media (PBS buffer, pH 7.4) and kept on an electromagnetic stirrer at 50 rpm and 37 ± 0.5 °C. Samples of 2 mL were taken at predetermined time intervals and replaced with equivalent volume of fresh media. The samples were then filtered (0.45 μm Chromafil^®^ syringe filter, Macherey-Nagel, Düren, Germany) and analyzed for drug content as mentioned above. Mean results of triplicate measurements and standard deviation were reported.

## 3. Results and Discussion

### 3.1. Synthesis and Characterization of Blank Casein Nanoparticles

Casein concentration and the crosslinking agent (CaCl_2_·2H_2_O, Mw = 147.01 g/mol) concentration were varied at three different levels according to the applied 3^2^ full factorial design. Three different concentrations of casein solution were used: low concentration 0.05% (1), medium concentration 0.1% (2) and high concentration 0.15% (3). The concentration of the crosslinking agent was also set at three levels: low concentration 0.5 M (1), medium concentration 1.0 M (2) and high concentration 1.5 M (3). The other process parameters were kept constant as described in the Materials and Methods section. The dependent variables were particle size and production yields. The composition of the obtained casein nanoparticles with different formulation variables is shown in Table 1.

**Figure 1:**
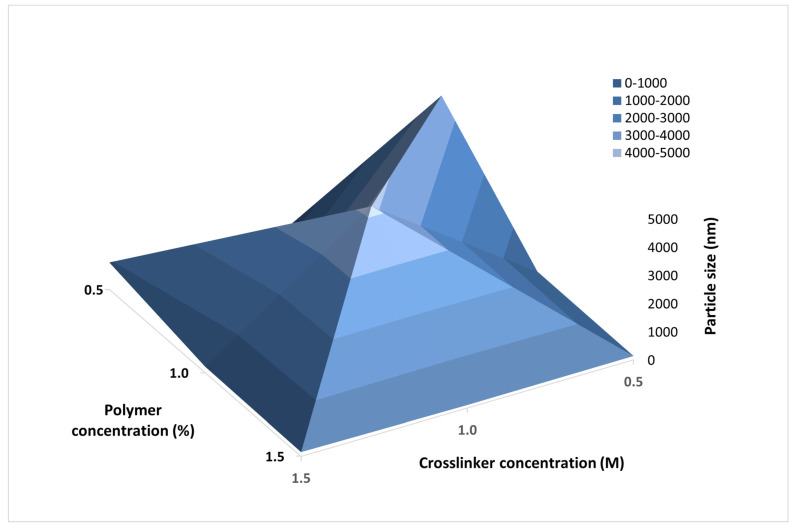
3D plot representing the impact of the concentration of the polymer and the crosslinking agent on the mean particle size of blank casein spray dried nanoparticles.

Nine batches of blank casein nanoparticles were obtained by spray drying technique. Mean particle sizes (Dv50, Table 1) varied in a wide range between 74.5 nm and 4.19 µm. The smaller particles showed extremely high degree of aggregation, leading to the formation of larger clusters, as evidenced from the scanning electron micrographs. Analysis of Dv10 was found to be far more representative for the particle size range of the formulated structures. According to the results, a tendency for reduction of the particle size was observed when the crosslinker concentration increased. According to the data published in the literature [42], the larger the amount of the crosslinker, the stronger the packing of the structure and the denser the micelle, resulting in particles of a smaller size range. Our results confirmed this relation, but the impact was not significant. As for the aggregation, clusters of nanoparticles occurred within each of the three groups of batches and no dependences could be outlined. For that reason, the combined effect of the two factors—the concentration of casein and the crosslinker—on the particle size was investigated, and the plot is shown in Figure 1. Since our goal was to produce particles of the smallest possible size, the batches revealing practically no or little degree of aggregation were considered for further investigation (samples prepared at casein concentration 1.5% and crosslinker concentration of 0.5 M proved to be unsatisfying).

**Figure 2:**
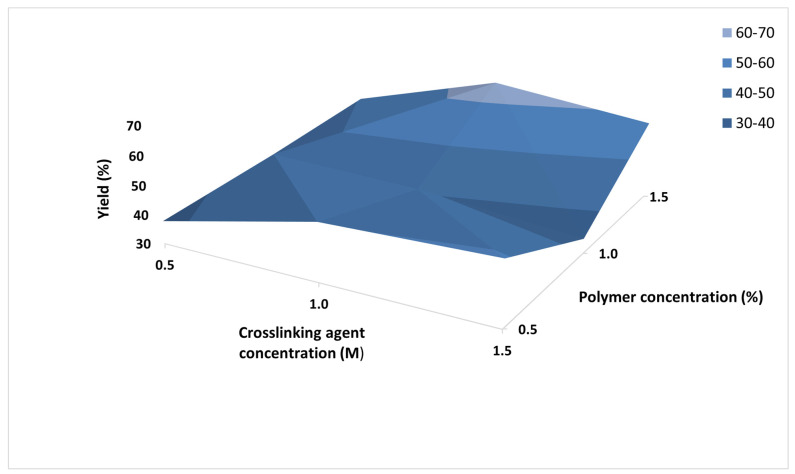
3D plot representing the impact of the concentration of the polymer (%) and the crosslinking agent (M) on the particles production yields.

Production yields, on the other hand, gradually increased when higher casein concentrations were used. The yields obtained varied in the range from 35.04% to 64.80%. Production yields were determinative for the selection of optimum models for drug loading and further investigation, therefore the combined effect of the two variables on the yields was studied. The plot is presented in Figure 2. The highest values were obtained when 1.5 M calcium chloride solution was used. Among all the formulated batches, Cas2-Ca3 (casein 1%, calcium chloride 1.5 M) was determined to be optimal in terms of production yield and desired particle size range.

### 3.2. Synthesis and Characterization of BZ Loaded Casein Nanoparticles

BZ-loaded CAS nanoparticles were prepared via coacervation method, followed by spray drying. In order to investigate the effect of polymer and drug concentration over the production yield, particle size, surface morphology, drug entrapment efficiency and release behavior, four batches of drug-loaded nanoparticles were prepared based on the optimized formulation of blank nanoparticles (sample Cas2-Ca3, prepared at 1.0% casein concentration, 1.5 M CaCl_2_·2H_2_O) and varying the polymer/drug ratio (1:1, 2:1, 4:1, 6:1) (Table 2). The results of the study are summarized in Table 3.

#### 3.2.1. Drug Loading and Entrapment Efficiency

Drug loading of the developed BZ-loaded casein nanoparticles varied in a wide range from 16.02% to 57.41%. A tendency for decrease in drug loading was observed with increase of polymer/drug ratio, which was not surprising regarding the amount of polymer used for the formulation of the model particles. Entrapment efficiency was substantial, varying from 76.23% to 78.82% for the samples prepared at 2:1, 4:1 and 6:1 ratio. Significantly lower entrapment efficiency was determined for the sample Cas2-Ca3-BZ-1, prepared at 1:1 polymer/drug ratio. It could be suggested that the polymer had a limited capacity to incorporate drug molecules during nanoparticles formulation. For the above sample, the amount of the polymer was probably not sufficient to entrap and retain the drug and form a stable structure.

Our hypothesis was confirmed by morphological analysis of the samples using scanning electron microscopy (Figure 4). The lack of clearly defined nanostructures in model Cas2-Ca3-BZ-1 was evidenced by the obtained scanning electron micrographs in contrast to the other samples. In addition, the larger amount of BZ in this sample probably led to displacement of calcium phosphate and disruption of micellar integrity. The phenomenon has been observed in other studies and has been thoroughly described in the literature [43]. With an increase of the polymer/drug ratio from 1:1 to 2:1, a double increase of the EE was observed (Table 3). Higher amounts of casein led to more efficient incorporation of benzydamine in the nanoparticles, which is probably due to the enhanced hydrophobic effect favoring micellar solubilization of the drug [19]. A further increase in the polymer/drug ratio (4:1 and 6:1) did not lead to a significant change in the drug entrapment efficiency.

#### 3.2.2. Production Yield

Production yields were high, ranging from 58.23% to 74.71% except for the batch produced at 1:1 polymer/drug ratio (34.61%). The increase in the amount of casein in the formulations, relative to BZ, led to a slight reduction of production yields, which was probably due to the enhanced viscosity of the feeding suspension, which made it difficult to pass through the spray mesh. On the other hand, batch Cas2-Ca3-BZ-1, although expected to provide the highest yield, refuted our suggestions. A possible explanation for this could be the disruption of micellar integrity due to displacement of calcium phosphate and the formation of precipitate prior to spray drying [44].

#### 3.2.3. Particle Size and Size Distribution

**Figure 3:**
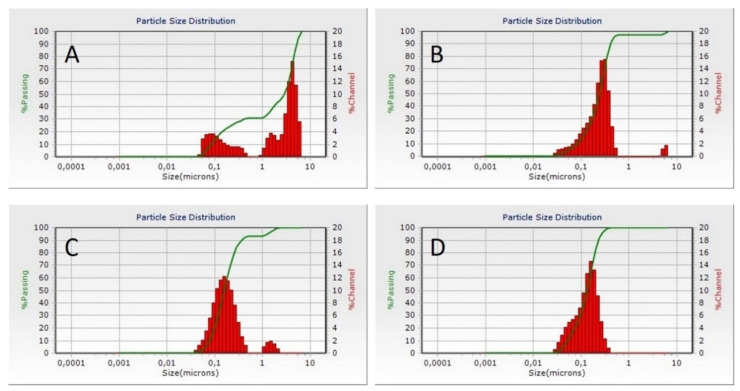
Dynamic light scattering histograms of BZ loaded casein nanoparticles of batches Cas2-Ca3-BZ-1 (**A**), Cas2-Ca3-BZ-2 (**B**), Cas2-Ca3-BZ-4 (**C**) and Cas2-Ca3-BZ-6 (**D**).

Particle size and size distribution were analyzed by dynamic light scattering and the results are presented in Table 3 and Figure 3. The median particle size ranged from 135.9 nm to 994.2 nm with a clear tendency for size reduction with increase in casein concentration. Bimodal particle size distribution was observed in batch Cas2-Ca3-BZ-1, suggesting a high aggregation tendency. However, no clearly distinguished structures were observed under scanning electron microscope, corresponding to the results obtained for production yield, drug loading and entrapment efficiency. Probably, nanoparticle formation could not be accomplished at 1:1 polymer/drug ratio, whereas the samples prepared at 2:1, 4:1 and 6:1 polymer/drug ratio were clearly distinguished and less cohesive, with minimal degree of aggregation.

#### 3.2.4. Surface Morphology

**Figure 4:**
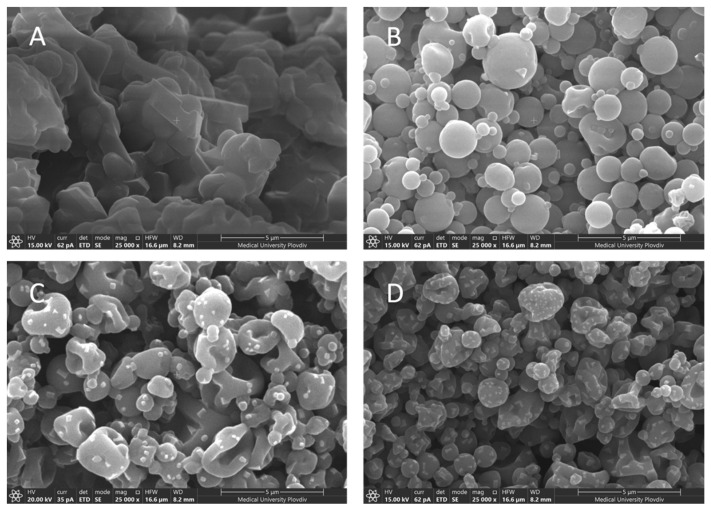
SEM micrographs of BZ-loaded casein nanoparticles of batches Cas2-Ca3-BZ-1 (**A**), Cas2-Ca3-BZ-2 (**B**), Cas2-Ca3-BZ-4 (**C**) and Cas2-Ca3-BZ-6 (**D**) at 25,000× magnification.

Surface morphology evaluation of the four batches of nanoparticles was performed using scanning electron microscopy. The micrographs are presented in Figure 4. Three different patterns of surface morphology were observed: rough spherical particles, wrinkled spherical particles and wrinkled irregularly shaped particles. A tendency towards increased surface roughness was observed with raising casein concentrations. Irregular, wrinkled, fragmented, and highly aggregated structures with an average particle size of about 994 nm were observed at drug/polymer ratio 1:1 (Figure 4A, batch Cas2-Ca3-BZ-1). It is well known that inlet temperature plays a key role in the spray drying process, significantly affecting the surface morphology of the dry particles. According to Both et al. [45], spray drying at high inlet temperatures generally results in the formation of less wrinkled particles with a large, hollow core. Therefore, it could be assumed that the higher viscosity of the feeding suspension together with the low inlet temperature (40 °C) might be associated with increased stickiness and subsequent agglomeration of these particles. As for the other three batches of nanoparticles, they all had a rounded shape and a wrinkled surface. In addition, as the concentration of the polymer raised and the percentage of drug diminished relative to the casein content, the rugosity degree of particles increased. It could be assumed that the lower drug content per unit mass led to the formation of loose matrix structures. Upon drying, these structures shrink, leading to the formation of smaller particles with multiple surface invaginations. Our hypothesis was confirmed by particle size analysis.

#### 3.2.5. Differential Scanning Calorimetry (DSC)

**Figure 5:**
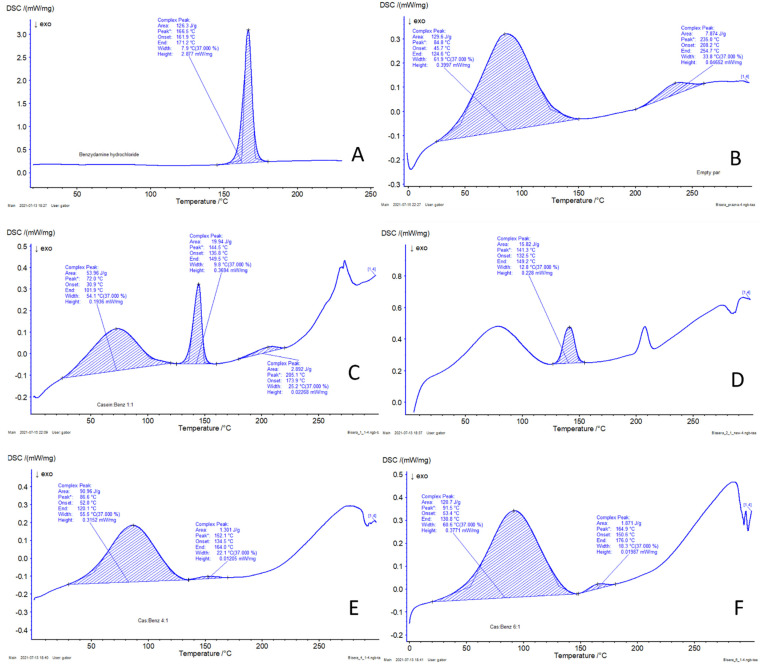
DSC thermograms of Benzydamine hydrochloride (**A**), blank casein nanoparticles (**B**), BZ-loaded casein nanoparticles of batches Cas2-Ca3-BZ-1 (**C**), Cas2-Ca3-BZ-2 (**D**), Cas2-Ca3-BZ-4 (**E**) and Cas2-Ca3-BZ-6 (**F**).

The phase state of BZ incorporated into the spray dried casein nanoparticles was analyzed using differential scanning calorimetry (DSC). The obtained thermograms are presented in Figure 5. The thermogram of casein revealed a broad endothermic peak at 84.8 °C, which could be attributed to the evaporation of water present in casein micelles. Benzydamine hydrochloride, on the other hand, being solid crystalline, showed a characteristic peak at 166.5 °C, which corresponds to its melting point. In drug-loaded samples, a gradual decrease in peak intensity was observed, with an increase in the polymer/drug ratio from 1:1 to 6:1, as shown in Figure 5C–F. It can be assumed that changes occurred in the degree of crystallinity of BZ during spray drying and the drug was partially transformed into its amorphous phase depending on the drug content in the formulated nanoparticles.

#### 3.2.6. Fourier-Transform Infrared Spectroscopy (FTIR)

**Figure 6:**
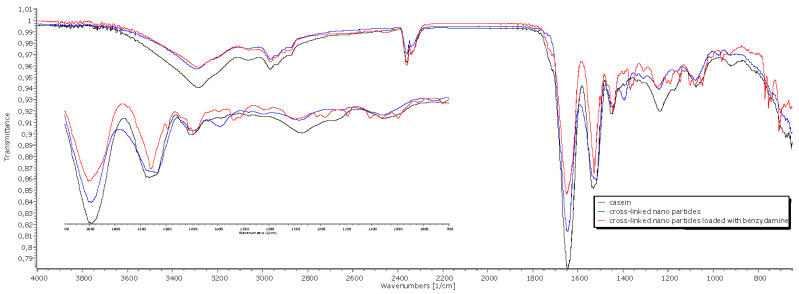
ATR-FTIR spectra of casein, crosslinked placebo nanoparticles and crosslinked BZ-loaded nanoparticles.

The spectra for casein (Figure 6) show peaks at 1646 cm^−1^ in the amide I region and 1530 cm^−1^ in the amide II region, which could be assigned to the stretching of the carbonyl group (C=O) and to the symmetric stretching of N-C=O bonds, respectively. Casein shows a band at 1077 cm^−1^, suggesting interactions of monocationic phosphates with Na^+^. The peaks in the amide I and amide II regions also appear in the crosslinked nanoparticles and in the drug loaded nanoparticles. The band at 977 cm^−1^, attributed to bionic phosphate, has very low intensity on the casein spectrum and increased intensity in the crosslinked systems, suggesting interaction with Ca^2^^+^. Characteristic bands for stretching of aromatic C=C at 1497 cm^−1^ of benzydamine hydrochloride can be seen in the spectra of the drug-loaded nanoparticles. The band at 1357 cm^−1^ is attributed to C-N vibrations of the heterocyclic ring.

#### 3.2.7. In Vitro Drug Release

**Figure 7:**
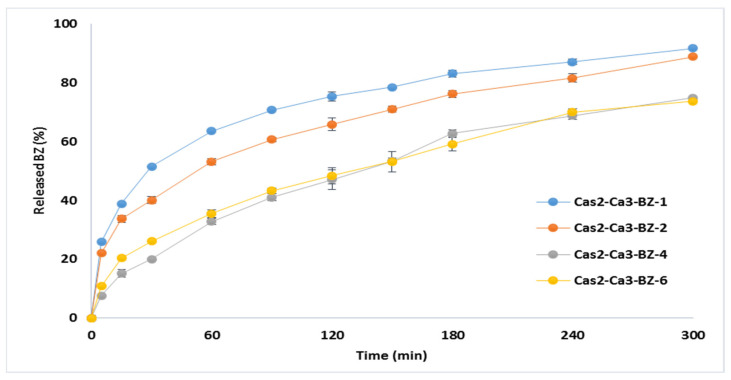
In vitro BZ release from spray dried casein nanoparticles prepared at different polymer/drug ratios (n = 3).

The dissolution profiles of BZ from casein nanoparticles are presented in Figure 7. The percentage of released drug during 5-h study was incomplete, varying from 73.30% (Cas2-Ca3-BZ-4) to 91.81% (Cas2-Ca3-BZ-1). Initial burst effect was observed in models Cas2-Ca3-BZ-1 and Cas2-Ca3-BZ-2 in the first 60 min, releasing more than 50% of the encapsulated drug. This was probably due to the higher drug loading and the accumulation of BZ in the periphery of the nanoparticles during the process of spray drying. The batches Cas2-Ca3-BZ-4 and Cas2-Ca3-BZ-6 demonstrated prolonged drug release over time, releasing almost 75% of the incorporated benzydamine. It was probably the greater amount of casein per unit mass in these two batches that refrained the drug from free diffusion from the particle core to the periphery, despite the larger surface area available for dissolution.

## 4. Conclusions

In this study, self-assembled casein nanocarriers were produced by nano spray drying. The process parameters were investigated, and an optimized model of blank casein nanostructures was outlined. Furthermore, four batches of BZ-loaded nanoparticles with a particle size from 135.9 nm to 994.2 nm were developed. BZ loading in the nanoparticles depended on the polymer/drug ratio. BZ was transformed from crystalline into amorphous during spray drying, which implies an increased dissolution rate. The drug release study confirmed the feasibility of the developed nanocarriers for prolonged delivery of benzydamine.

## Figures and Tables

**Table 1:** Composition and characteristics of blank casein nanoparticles (n = 3, PDI (polydispersity index), Dv10, Dv50 and Dv90 (10, 50 and 90% of the total volume of particles, respectively, are with size below the indicated value).

Sample Code	Variables		Dv10 ± SD (nm)	Dv50 ± SD (nm)	Dv90 ± SD (nm)	PDI	ζ ± SD (mV)	Yeild ± SD (%)
	Polymer (%)	Crosslinker (M)						
Cas1-Ca1	0.5	0.5	2885.0 ± 5.26	3470.0 ± 22.3	4020.0 ± 6.6	109.20	−15.4 ± 0.4	37.87 ± 9.26
Cas1-Ca2	0.5	1.0	48.5 ± 2.04	174.5 ± 4.1	256.1 ± 2.7	8.44	−22.5 ± 0.8	43.54 ± 2.04
Cas1-Ca3	0.5	1.5	46.1 ± 7.25	133.2 ± 1.6	2156.0 ± 4.7	34.47	−19.0 ± 0.6	50.30 ± 7.25
Cas2-Ca1	1.0	0.5	89.8 ± 2.83	138.1 ± 3.5	463.2 ± 3.0	22.68	−17.0 ± 0.7	49.57 ± 2.83
Cas2-Ca2	1.0	1.0	51.3 ± 4.55	4190.0 ± 20.2	6050.0 ± 3.2	543.90	−13.3 ± 0.7	40.88 ± 4.55
Cas2-Ca3	1.0	1.5	36.5 ± 3.02	104.1 ± 8.5	191.0 ± 9.0	1.21	−23.6 ± 0.6	64.80 ± 3.02
Cas3-Ca1	1.5	0.5	91.8 ± 2.57	956.2 ± 14.3	5556.0 ± 5.1	6.18	−17.9 ± 0.7	51.04 ± 2.57
Cas3-Ca2	1.5	1.0	115.0 ± 4.50	212.9 ± 2.9	1362.0 ± 7.9	8.06	−15.1 ± 0.5	35.04 ± 4.50
Cas3-Ca3	1.5	1.5	90.1 ± 2.69	149.2 ± 2.6	2944.0 ± 4.0	13.21	−16.3 ± 0.5	57.68 ± 2.69

**Table 2:** Composition of BZ-loaded casein nanoparticles of various batches.

Sample Code	Sodium Caseinate (%)	Benzydamine HCl (mg)	Variables	
			Polymer: Drug Ratio	
Cas2-Ca3-BZ-1	1.5	1.500	1	1
Cas2-Ca3-BZ-2	1.5	0.750	2	1
Cas2-Ca3-BZ-4	1.5	0.375	4	1
Cas2-Ca3-BZ-6	1.5	0.250	6	1

**Table 3:** Characteristics of the spray dried BZ-loaded casein nanoparticles (n = 3). DL = drug loading, EE = entrapment efficiency.

Sample Code	Particle Size ± SD (nm)	ζ ± SD (mV)	DL ± SD (%)	EE ± SD (%)	Yield ± SD (%)
Cas2-Ca3-BZ-1	994.2 ± 2.21	18.11 ± 0.86	57.41 ± 0.27	34.61 ± 0.23	30.42 ± 4.28
Cas2-Ca3-BZ-2	243.6 ± 2.47	16.33 ± 0.55	35.04 ± 034	78.82 ± 0.39	74.71 ± 5.41
Cas2-Ca3-BZ-4	159.8 ± 2.43	15.24 ± 0.58	26.21 ± 0.22	76.23 ± 0.28	68.76 ± 5.01
Cas2-Ca3-BZ-6	1359 ± 1.73	14.23 ± 0.66	16.02 ± 0.31	77.44 ± 0.57	58.23 ± 5.08

## Data Availability

The data presented in this study are available on request from the corresponding author.
